# Impact of Nutrition Education on Dietary Intake and Body Composition Among Czech University Students Studying Nutrition and Food

**DOI:** 10.3390/healthcare14101258

**Published:** 2026-05-07

**Authors:** Anna Jílková, Diana Chrpová, Adam Hruška, Andrea Maťhová, Lenka Kouřimská

**Affiliations:** 1 Faculty of Agrobiology, Food and Natural Resources, Czech University of Life Sciences Prague, 16521 Prague, Czech Republic; chrpovad@af.czu.cz (D.C.); mathova@af.czu.cz (A.M.); kourimska@af.czu.cz (L.K.); 2 Faculty of Tropical AgriSciences, Czech University of Life Sciences Prague, Kamýcká 129, 16500 Prague, Czech Republic; hruskaa@af.czu.cz

**Keywords:** nutrition education, dietary intake, body composition, bioelectrical impedance analysis, food records, micronutrients, young adults, preventive healthcare

## Abstract

**Highlights:**

**What are the main findings?**

- Nutrition education significantly improved several dietary variables among university nutrition students.
- Short-term dietary improvements did not result in measurable changes in body composition.

**What are the implications of the main findings?**

- Nutrition education may improve diet quality among young adults.
- Preventive strategies within university settings may promote healthier lifestyle behaviour.

**Abstract:**

**Background/Objectives:** University students frequently exhibit suboptimal dietary habits, and even those enrolled in nutrition-related programmes may fail to meet recommended intakes of several key nutrients. This study aimed to assess changes in dietary intake and body composition over a single academic semester among university nutrition students. **Methods:** A prospective pre–post study was conducted with 102 students at the Czech University of Life Sciences Prague. Dietary intake was assessed using a 3-day food record and evaluated for energy, macronutrients, and specific micronutrients. Body composition was measured by a multi-frequency bioelectrical impedance analysis. Changes between baseline and follow-up were analysed using paired statistical tests with the false discovery rate correction. Predictors of follow-up body fat percentage were examined using an analysis of covariance. **Results:** At baseline, mean daily energy intake was 2114 ± 632 kcal. A particularly low intake was observed for dietary fibre (15.45 ± 8.46 g/day), potassium (2013 ± 954 mg/day), iodine (63.5 ± 69.8 µg/day), and vitamin D (2.31 ± 3.01 µg/day), whereas protein intake was relatively high. During follow-up, significant increases were observed in the intake of carbohydrates (+54.2 g/day), dietary fibre (+9.3 g/day), potassium (+766 mg/day), vitamin C (+69.2 mg/day), and magnesium (+86.2 mg/day), together with lower sodium and saturated fat intake (all adjusted *p* < 0.001). No significant short-term changes were found in body weight, body fat percentage, or skeletal muscle mass. Follow-up body fat percentage was primarily associated with baseline adiposity. **Conclusions:** One semester of nutrition-related education was associated with improved dietary intake, particularly for fibre and selected micronutrients, but not with measurable short-term changes in body composition. These findings suggest that nutrition education may support healthier dietary behaviour and may contribute to preventive healthcare strategies in young adults.

## 1. Introduction

Dietary habits established during young adulthood play a critical role in shaping long-term health outcomes, including body weight regulation, cardiometabolic risk, and overall nutritional status. University students are considered a nutritionally vulnerable population, frequently exhibiting suboptimal diet quality and insufficient intake of key nutrients. The dietary fibre intake among university students has been reported to average approximately 17.8 g/day, which does not meet the recommended intake levels [1]. Similarly, population-based data from European countries indicate that dietary fibre intake remains below recommendations for most individuals, with 75–90% of young adults failing to meet the recommended daily intake [2].

These findings suggest that even populations with potential access to nutritional knowledge may fail to meet basic dietary recommendations, highlighting the gap between knowledge and actual dietary behaviour. The transition to university life is often accompanied by increased independence in food choices, irregular daily schedules, academic stress, and changes in physical activity patterns, all of which may negatively affect diet quality. In addition, students’ dietary behaviour may also be influenced by their financial situation, as limited financial resources can constrain food choice, meal regularity, and access to nutritionally balanced food [3,4,5]. As a result, the university environment has been widely recognised as an important setting for health promotion and preventive interventions aimed at improving dietary habits among young adults. Several reviews indicate that university-based programmes can positively influence nutrition knowledge and selected dietary behaviours, although the magnitude and consistency of these effects vary across studies [6,7,8,9].

Students enrolled in nutrition- and food-related degree programmes represent a unique subgroup within the university population. Given their academic training, they are expected to possess greater nutrition knowledge than students from other disciplines. However, accumulating evidence suggests that greater knowledge does not automatically translate into healthier dietary behaviour. Recent literature highlights a persistent gap between the nutrition knowledge and actual food intake, even among nutrition students, with inconsistent associations reported across studies depending on the assessment tools and dietary indicators used [10,11]. These findings emphasise the importance of evaluating whether structured nutrition education embedded within university curricula leads to measurable improvements in real-life dietary intake rather than improvements in theoretical knowledge alone [3]. Understanding this relationship is particularly relevant from a public health perspective, as nutrition students will later play an important role in promoting healthy dietary behaviours among the general population.

Assessing dietary intake in free-living individuals remains methodologically challenging. Self-reported dietary assessment methods, including food frequency questionnaires, 24 h recalls, and food records, are subject to both random and systematic measurement errors. Methodological reviews have consistently shown that misreporting—particularly the underreporting of energy intake—is common across self-reported methods, even in well-educated populations [12,13]. Despite these limitations, the food records remain widely used in intervention and educational research because they provide detailed prospective information on foods, beverages, and portion sizes and allow within-person comparisons when standardised protocols are applied [12,14]. Short-term dietary records, such as three-day food logs including both weekdays and a weekend day, are therefore considered suitable for detecting changes in dietary intake over relatively short follow-up periods, particularly in pre–post study designs [15,16,17].

In addition to dietary intake, body composition assessment provides complementary insights into the potential physiological relevance of dietary changes. Bioelectrical impedance analysis (BIA) is frequently used in clinical and field research settings due to its non-invasive nature, ease of use, and suitability for repeated measurements. Multi-frequency, segmental BIA devices have demonstrated high test–retest reliability for key body composition parameters, including fat mass, fat-free mass, and visceral fat [18,19]. However, validation studies comparing BIA with reference methods such as dual-energy X-ray absorptiometry (DXA) or four-compartment models have reported a systematic bias that varies according to sex, body composition, and hydration status [20]. Consequently, BIA is primarily recommended for monitoring changes over time within individuals or groups under standardized conditions rather than for the absolute quantification of body compartments. Due to its practicality, greater affordability, and better accessibility compared to DXA, BIA is well-suited for use in university settings, as it does not require highly specialized equipment or extensive examiner training [21].

Taken together, the existing evidence supports the potential of university-based nutrition education to improve dietary behaviours and promote healthier lifestyles among young adults. However, important gaps remain in longitudinal research, particularly in studies that combine repeated dietary assessments with repeated body composition measurements within a defined educational period. This gap is especially pronounced among nutrition students, whose dietary behaviours are relevant not only to personal health but also to their future professional roles in health promotion and disease prevention. Understanding whether nutrition education during university studies leads to measurable improvements in dietary intake and favourable changes in body composition may therefore contribute to the development of more effective educational strategies aimed at improving health-related behaviours [7].

Currently, however, longitudinal data on dietary intake, nutritional status, and body composition among Czech university students—particularly nutrition students—remain limited. Existing studies in the Czech Republic have primarily focused on cross-sectional assessments of body composition and lifestyle factors among student populations, highlighting differences in adiposity and physical activity patterns among young adults [22]. Therefore, the present study aimed to evaluate changes in dietary intake assessed by repeated three-day dietary records and changes in body composition measured by multi-frequency bioelectrical impedance analysis among students of nutrition at the Czech University of Life Sciences Prague over the course of one academic semester following structured nutrition education. We hypothesized that the nutrition education would lead to improvements in dietary quality but only minimal short-term changes in body composition.

## 2. Materials and Methods

### 2.1. Study Design

This study was designed as a prospective longitudinal observational study conducted over approximately three months, spanning one academic semester. Data collection was conducted at two time points: the baseline at the beginning of the semester and the follow-up at the end of the semester. A within-subject longitudinal design was used to capture short-term changes in dietary intake and body composition during a defined period of nutrition education while minimizing inter-individual variability [23].

The study was conducted in accordance with the ethical principles of the Declaration of Helsinki and relevant institutional regulations. Ethical approval was obtained from the Ethics Committee of the Czech University of Life Sciences Prague (File reference number 01/2024; 18 April 2024). All procedures were non-invasive and ensured participant anonymity. Before participation, students were informed about the study procedures and provided written informed consent. Data handling complied with applicable legal regulations and institutional data protection policies.

### 2.2. Participants

Participants were undergraduate students enrolled in a nutrition-related study programme at the Czech University of Life Sciences Prague, predominantly in their third year of study. The inclusion criteria were as follows: active enrolment in the selected course, age between 18 and 27 years, submission of signed informed consent, and fulfilment of standard preconditions for bioelectrical impedance analysis (BIA) measurements.

The exclusion criteria included pregnancy, any changes in medication during the study period that could affect body composition or hydration status, significant health complications (e.g., injury or illness affecting physical activity), and discontinuation of university studies during the observation period. Participants were also excluded if they had contraindications to BIA measurements, such as implanted electronic medical devices (e.g., pacemakers), acute illness at the time of measurement, or inability to comply with standardized pre-measurement instructions [24,25].

Only participants who completed both dietary assessments and body composition measurements at baseline and follow-up were included in the final analysis.

### 2.3. Baseline Anamnesis and Lifestyle Assessment

Baseline demographic and lifestyle information was collected using a structured questionnaire administered upon enrolment. The survey gathered details regarding age, sex, personal medical history, and family history of specific chronic diseases, and key lifestyle factors.

Lifestyle variables included smoking status, alcohol consumption, dietary supplements use, dietary patterns (e.g., vegetarianism), and the presence of any food allergies or intolerances. Participants also reported their typical meal frequency, daily fluid intake, and primary eating environment (e.g., home-prepared meals versus university catering services).

Physical activity was assessed using self-reported measures, including activity type and typical weekly frequency. Such measures are commonly used in observational studies when objective monitoring is not feasible [26].

### 2.4. Dietary Assessment

Dietary intake was assessed using repeated three-day dietary records collected at baseline and follow-up. Each dietary record included two weekdays and one weekend day to better capture habitual intake and day-to-day variability among free-living individuals [27,28].

Participants were instructed to document all food and beverage consumption, including portion sizes and preparation methods. Standardized instructions were provided to improve the accuracy and completeness of the records.

The dietary data were analyzed using NutriServis PROFI (https://www.nutriservis.cz/) (NutriServis, Prague, Czech Republic), a software application commonly used in Czech academic and clinical nutrition settings. The analysis provided estimates of daily energy intake (kcal and kJ), macronutrient intake (protein, fat, and carbohydrates), and selected micronutrient intake, including dietary fibre, calcium, iron, vitamin D, zinc, potassium, and sodium.

Nutritional intakes were compared with relevant D-A-CH reference values for nutrient intake [29]. These reference values were used to evaluate the adequacy of nutrient intake within the study population.

### 2.5. Body Composition Assessment

Body composition was assessed using multi-frequency segmental bioelectrical impedance analysis (MF-BIA) with the InBody 770 device (InBody Co., Ltd., Seoul, Republic of Korea). This analyser uses an eight-point tactile electrode system and performs segmental impedance measurements across five body regions (right arm, left arm, trunk, right leg, and left leg).

Multi-frequency BIA is widely used in research settings due to its non-invasive nature, feasibility, and suitability for repeated measurements, although its accuracy may be affected by participants’ hydration status and recent behaviours. Measurements were conducted under standardised conditions in accordance with manufacturer recommendations [20,21].

Primary body composition outcomes included body weight, body mass index (BMI), fat mass (kg and %), fat-free mass (kg), skeletal muscle mass, visceral fat area (cm^2^), and total body water. Body height was measured using a SECA 213 stadiometer (SECA, Hamburg, Germany). Participants were assessed approximately three months apart, at the beginning and end of the semester, during two recurring periods each year (October–December and February–April), concurrently with dietary record collection.

### 2.6. Educational Exposure During the Semester

During the observation period, participants attended a structured university curriculum in human nutrition as part of their last year of academic programme. The courses were completed within one semester (approximately 5–6 weeks) and included lectures (2 h/week) and practical seminars (1–2 h/week), combining theoretical instruction with applied training.

The curriculum included key courses focused on human nutrition. Food Product Knowledge explored relationships between food groups, their processing, and their role in human nutrition, including their nutritional value and relevance to health. Nutrients and Nutrient Needs of Humans provided fundamental knowledge on nutrient functions, digestion and absorption, nutrient requirements, and the effects of deficiency and toxicity.

Rational Nutrition and Basics of Dietetics focused on principles of a balanced diet, nutritional requirements of specific population groups, and evaluation of dietary patterns, including therapeutic diets. Practical seminars included dietary assessment, meal planning, case study analysis, and the use of dietary analysis software. Nutrition in Health and Disease covered dietary approaches in disease prevention and management, with practical sessions aimed at developing the ability to interpret scientific evidence and critically evaluate nutrition-related information.

The programme also included practical training in a nutrition counselling setting (minimum 25 h). Students worked with real clients and applied acquired knowledge in real-life situations, including assessing dietary intake and nutritional status.

All participants included in the analysis completed the relevant semester of the curriculum; those who did not were excluded from the final analysis. The educational exposure was not designed as a controlled intervention but reflects a real-life academic learning environment with the potential to influence dietary behaviour. These courses were delivered by qualified nutrition professionals, including registered dietitians, and represent a structured educational exposure that could plausibly influence students’ dietary awareness and everyday dietary behaviours during the semester [7,30].

### 2.7. Statistical Analysis

Statistical analyses were performed using Python (Python Software Foundation, version 3.x, Wilmington, DE, USA) and Microsoft Excel (Microsoft Corp., Redmond, WA, USA). Continuous variables are presented as mean ± standard deviation (SD) for normally distributed data or as median and interquartile range (IQR) for non-normally distributed variables. Given the adoption of a Bayesian modelling framework, formal normality testing of distributional assumptions was addressed directly within the probabilistic models. To evaluate the effectiveness of the educational intervention, changes in dietary intake and body composition were analysed using Bayesian hierarchical (multilevel) models with partial pooling. These models explicitly account for the repeated-measures structure of the data (baseline and follow-up nested within individuals) and allow simultaneous estimation of both population-level effects and individual-specific responses. For each outcome, a longitudinal hierarchical model was specified with participant-specific random intercepts and random slopes for time (pre–post change), enabling estimation of individual trajectories. Individual-level effects were modelled as draws from a population distribution.

Baseline covariates (age, sex, and baseline value of the outcome) were incorporated as fixed effects, and their interactions with time were included to assess effect modification. Sex was therefore treated as a covariate within the hierarchical structure rather than analysed solely via stratified comparisons, mitigating issues arising from unequal group sizes. Model parameters were estimated using Markov chain Monte Carlo (MCMC) sampling, and results are reported as posterior distributions summarized by means and 95% credible intervals. Convergence was assessed using standard diagnostics, including the potential scale reduction factor and visual inspection of trace plots. In contrast to traditional null hypothesis significance testing, inference was based on the magnitude and uncertainty of estimated effects, as well as posterior probabilities of direction (e.g., probability of improvement).

To characterise inter-individual variability in response to the intervention, participant-specific posterior estimates of change (random slopes) were extracted. Based on these distributions, individuals were classified as responders, non-responders, or uncertain responders according to the posterior probability of a beneficial effect (threshold ≥ 0.80). Furthermore, the variability (standard deviation) of individual-level effects was quantified to assess heterogeneity of treatment response at the population level (Supplementary Materials).

Associations between dietary changes and body composition were examined using the analysis of covariance (ANCOVA), adjusting for baseline body fat percentage, age, sex, and changes in energy and protein intake. Sex was included as a covariate rather than a primary factor due to an imbalance in group sizes. Where appropriate, exploratory sex-stratified analyses were conducted; however, these were interpreted descriptively due to limited statistical power in the male subgroup. Interaction terms (sex × dietary change) were tested selectively to assess potential effect changes but were not the primary focus of inference. Given the exploratory nature of the analysis, only selected dietary variables and body composition outcomes were included in the ANCOVA models, based on their physiological relevance and the study’s main findings. Statistical significance was set at *p* < 0.05.

## 3. Results

### 3.1. Participants and Study Flow

A total of 198 students enrolled in the Nutrition and Food programme at the Czech University of Life Sciences Prague were initially assessed for participation. Of these, 96 participants were excluded from the final analysis due to incomplete datasets. Reasons for exclusion included incomplete dietary records (*n* = 42), missing body composition measurements (*n* = 23), and voluntary withdrawal from dietary or anthropometric assessment (*n* = 31).

**Figure 1:**
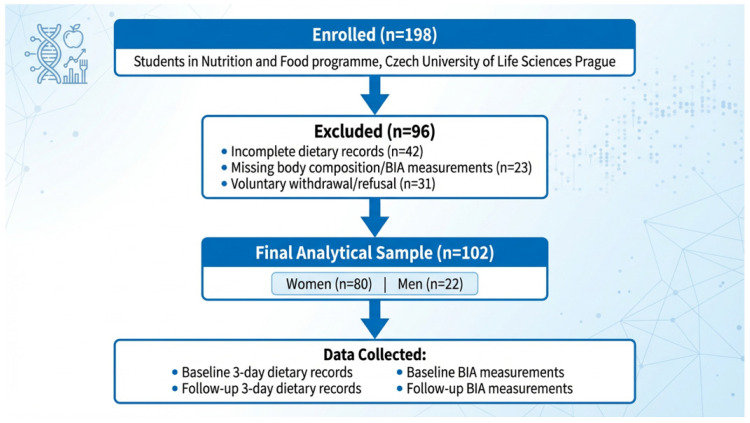
The study flow of the participants.

The final analytical sample consisted of 102 participants, including 80 women and 22 men, who completed both baseline and follow-up assessments and provided evaluable dietary records. The flow of participants through the study is illustrated in Figure 1.

### 3.2. Characteristics of the Baseline Cohort

Baseline demographic and lifestyle characteristics are presented for the broader baseline cohort (*n* = 198), for whom questionnaire data were available. Participants were excluded due to incomplete dietary records, missing body composition measurements, inability to attend the second assessment, or personal reasons (e.g., study discontinuation or study abroad). Longitudinal analyses of dietary intake and body composition were performed on an analytical sample of 102 participants.

The cohort consisted predominantly of women (83.3%, *n* = 165), while men represented 16.7% (*n* = 33) of participants. Most students reported good overall health (59.1%, *n* = 117). The most frequently reported health conditions included disordered eating or a history of eating disorders (8.6%, *n* = 17), gastrointestinal complaints (7.1%, *n* = 14), and thyroid disorders (3.5%, *n* = 7), while diabetes mellitus was rare (0.5%, *n* = 1).

Family history revealed a relatively high prevalence of cardiometabolic conditions among parents. Paternal hypertension was reported by 28.8% of students (*n* = 57) and maternal hypertension by 23.7% (*n* = 47). Parental overweight or obesity was reported in approximately 15.2% of parents (*n* = 30), hypercholesterolemia in 16.2% (*n* = 32), and type 2 diabetes in 7.6% (*n* = 15).

Regarding lifestyle habits, most students reported primarily consuming home-prepared meals (80.8%, *n* = 160), while approximately one-fifth regularly used institutional catering (19.2%, *n* = 38). Nearly half of the participants reported part-time employment (45.5%, *n* = 90).

Food allergies or intolerances were reported by several students, most commonly lactose intolerance (11.1%, *n* = 22). 7.1% of participants (*n* = 14) reported vegetarian dietary patterns. Dietary supplement use was also common, particularly magnesium (18.7%, *n* = 37), omega-3 fatty acids (11.6%, *n* = 23), and zinc (9.6%, *n* = 19).

Most students reported regular physical activity, typically three to five sessions per week, with fitness training (49.0%, *n* = 97) and walking or hiking (39.4%, *n* = 78) being the most common activities.

The most common personal goal related to body composition was weight maintenance (56.6%, *n* = 112), followed by fat reduction (25.3%, *n* = 50) and muscle gain (9.1%, *n* = 18).

### 3.3. Baseline Dietary Intake

Baseline dietary intake of the analytical sample is presented in Table 1, including comparisons between women and men, as well as the proportion of participants meeting the D-A-CH dietary reference values. Mean daily energy intake was 2114 ± 632 kcal (8845 ± 2644 kJ), with average intakes of 107.2 ± 45.1 g of protein, 80.7 ± 26.6 g of fat, and 241.4 ± 69.6 g of carbohydrates. Dietary fibre intake was relatively low (15.5 ± 8.5 g/day).

Men showed significantly higher intakes of several nutrients compared with women. Specifically, males had a higher intake of energy, protein, fat, carbohydrates, cholesterol, sodium, potassium, magnesium, zinc, iodine, and vitamin C (*p* < 0.05). These differences largely reflect the higher total energy intake among men. Given the smaller number of male participants, these differences should be interpreted with caution and are more likely to reflect differences in total energy intake than sex-specific dietary patterns per sex. Furthermore, regardless of these differences, the overall dietary pattern observed in both sexes was similar.

When compared with D-A-CH dietary reference values, several nutrients showed relatively low mean intake levels in the study population. For example, mean intakes of dietary fibre (15.5 ± 8.5 g/day), potassium (2013 ± 954 mg/day), iodine (63.51 ± 69.79 µg/day), and vitamin D (2.31 ± 3.01 µg/day) were below the recommended values.

In contrast, protein intake exceeded the recommended levels in the study population. In addition to mean intake values, Table 1 also presents nutrient intake in women and men as a percentage of the corresponding D-A-CH reference values, providing insight into the extent to which recommended intake levels were achieved across the cohort.

### 3.4. Changes in Dietary Intake During the Semester

Changes in dietary intake during the semester were evaluated using both conventional group-level analyses and Bayesian hierarchical models that account for participant-specific trajectories. Changes in dietary intake between baseline and follow-up are presented in Table 2.

At the group level, the largest increases were observed in carbohydrate intake (+54.2 g/day; Cohen’s d = 1.07; FDR-adjusted *p* < 0.001) and dietary fibre intake (+9.33 g/day; d = 1.04; *p* < 0.001). Significant increases were also found for several micronutrients, including potassium (+766 mg/day; d = 0.95; *p* < 0.001), vitamin C (+69.2 mg/day; d = 0.86; *p* < 0.001), and magnesium (+86.2 mg/day; d = 0.73; *p* < 0.001). Moderate but statistically significant increases were observed for iodine, zinc, vitamin K, vitamin E, and iron (all FDR-adjusted *p* < 0.05). In contrast, sodium intake (−221 mg/day; d = −0.26; *p* = 0.011) and saturated fat intake (−2.04 g/day; d = −0.24; *p* = 0.019) decreased, suggesting an overall shift toward a healthier dietary profile.

While these results indicate substantial average improvements, they do not capture interindividual variability. To address this, Bayesian hierarchical models were used to estimate individual-level changes while accounting for partial pooling across participants. For protein intake, the Bayesian model indicated a positive average change over time (population mean time effect: 0.165 SD units, 95% credible interval [0.035, 0.271]). However, substantial heterogeneity in individual responses was observed (SD of individual time effects: 0.554 SD units). At the participant level, 59 individuals were classified as responders, 37 as worseners, and 5 as uncertain based on posterior probabilities, with a median probability of benefit of 0.954. These findings indicate that, despite a positive average trend, a considerable proportion of participants did not exhibit beneficial changes.

Model-based interaction effects suggested that changes in protein intake were associated with participant characteristics. The time-by-sex interaction was positive (β = 0.586, 95% HDI [0.290, 0.892]), indicating that male participants tended to show larger increases compared with females. In contrast, the time-by-baseline interaction was negative (β = −0.447, 95% HDI [−0.591, −0.327]), suggesting that participants with higher baseline protein intake exhibited smaller improvements. The interaction between age and time was small and uncertain (β = 0.049, 95% HDI [−0.054, 0.147]).

For mean energy intake, the Bayesian model indicated a decrease over time (population mean time effect: −0.175 SD units, 95% credible interval [−0.225, −0.109]), again accompanied by substantial interindividual variability (SD of individual time effects: 0.302 SD units). Interaction effects suggested that changes in energy intake were associated with age (β = 0.073, 95% HDI [0.023, 0.126]), sex (β = 0.699, 95% HDI [0.568, 0.844]), and baseline energy intake (β = −1.315, 95% HDI [−1.379, −1.260]). The results indicate that the magnitude of change differed across subgroups, with participants’ baseline characteristics influencing their response to the intervention.

Exploratory sex-stratified analyses revealed a significant interaction between sex and MUFA intake (*p* = 0.0037), with males showing a greater increase than females. However, given the limited sample size of the male subgroup, this finding should be interpreted with great caution and requires confirmation in larger cohorts. No other nutrient changes differed significantly by sex (all *p* > 0.05), suggesting that semester-related dietary changes were broadly similar across sexes, although these analyses were underpowered.

### 3.5. Baseline Body Composition Characteristics and Body Composition Changes

Baseline body composition characteristics of the participants are presented in Table 3. The mean age of the sample was 22.7 ± 1.5 years, with no significant difference between women and men (*p* = 0.366). The overall mean BMI was 23.5 ± 3.1 kg/m^2^, corresponding to the normal weight range. Four women exceeded the BMI threshold for obesity (≥30.0 kg/m^2^) and simultaneously exhibited an extremely high body fat percentage (>39%). In addition, four women had a BMI in the underweight range (<18.5 kg/m^2^).

Significant sex-related differences were observed in several anthropometric and body composition parameters. Male participants were significantly taller and heavier and had a higher BMI than female participants (all *p* < 0.001). Men also exhibited substantially greater skeletal muscle mass, total body water, bone mineral content, and basal metabolic rate than women (all *p* < 0.001).

Conversely, women had significantly higher body fat percentage and visceral fat area compared with men (*p* ≤ 0.001). No significant difference between sexes was observed in the waist–hip ratio (*p* = 1.000). Although statistical significance is limited by the unequal sex distribution, the findings reflect typical sex-specific differences in body composition, with higher lean mass in men and higher relative adiposity in women.

Changes in body composition during the study period were re-evaluated using Bayesian hierarchical models that account for participant-specific trajectories. On the standardized (z-score) scale, the average changes were small across all outcomes. Body weight showed a slight decrease over time (population mean time effect: −0.016 SD units, 95% credible interval [−0.020, −0.011]). In contrast, skeletal muscle mass increased modestly (0.021 SD units, 95% CrI [0.016, 0.027]), while body fat mass also showed a small increase (0.012 SD units, 95% CrI [0.000, 0.024]), indicating a slight unfavourable shift in adiposity on average. The InBody score exhibited a small positive change; however, the corresponding credible interval included zero (0.023 SD units, 95% CrI [−0.002, 0.047]), suggesting that this change was uncertain.

Despite the small average effects, substantial interindividual variability in change trajectories was observed. The variability in individual time effects was largest for body fat mass (SD = 0.281), followed by InBody score (SD = 0.237), body weight (SD = 0.173), and skeletal muscle mass (SD = 0.105). These findings indicate that, although mean changes at the group level were minimal, participants responded heterogeneously, with differing directions and magnitudes of change across individuals.

### 3.6. Associations Between Dietary Changes and Body Composition

Associations between dietary changes and follow-up body fat percentage were evaluated using ANCOVA models adjusted for baseline body fat percentage, age, sex, and changes in energy and protein intake. Statistical significance was set at *p* < 0.05.

In the primary ANCOVA model (*n* = 102), baseline body fat percentage was the strongest predictor of follow-up body fat percentage (β = 0.927, SE = 0.031, *p* < 0.001). The model explained 94.3% of the variance in body fat percentage (adjusted R^2^ = 0.940). Changes in protein intake (β = −0.005, *p* = 0.599) and energy intake (β = 0.0006, *p* = 0.420) were not significantly associated with follow-up body fat percentage. Age and sex were also non-significant covariates (both *p* > 0.10), although the ability to detect sex effects was limited by the smaller number of male participants (Table 4).

Additional analyses examining potential interaction and nonlinear effects did not improve model fit. Including an interaction term for baseline body fat percentage × change in protein intake was not significant (*p* = 0.791), and adding a quadratic term for baseline body fat percentage did not indicate nonlinearity (*p* = 0.823).

Further regression models predicting changes in body fat percentage explained only a small proportion of the variance (R^2^ = 0.080; overall *p* = 0.138), suggesting that dietary changes during the observation period were not strong predictors of changes in body fat.

Sex-stratified analyses showed no significant association between changes in protein intake and changes in body fat percentage in either females (*n* = 80; R^2^ ≈ 0.000; *p* = 0.966) or males (*n* = 22; R^2^ = 0.009; *p* = 0.671). Given that these analyses were substantially underpowered in males, the results should be interpreted as descriptive rather than confirmatory.

## 4. Discussion

The present study evaluated whether one semester of nutrition-related academic exposure was associated with changes in dietary intake and body composition among Czech university students enrolled in a nutrition-focused programme and assessed their nutritional status. Several main findings emerged. First, despite studying nutrition, participants showed several baseline nutritional inadequacies, especially in dietary fibre, vitamin D, potassium, iodine, and calcium intake, while protein intake was relatively high. Second, during the semester, significant improvements were observed in several dietary parameters, including higher intake of carbohydrates, fibre, potassium, vitamin C, magnesium, iodine, zinc, vitamin K, vitamin E, iron, and monounsaturated fatty acids, together with lower sodium and saturated fat intake. Third, these favourable dietary changes were not accompanied by significant short-term changes in body composition, and follow-up body fat percentage appeared to be influenced mainly by baseline adiposity rather than by changes in reported energy or protein intake.

The baseline findings are important because they show that even students receiving formal nutrition education may fail to meet recommended intakes of several key nutrients. This pattern is consistent with broader evidence suggesting that university students often show suboptimal diet quality, irregular eating habits, insufficient intake of key micronutrients, eating disorders, and body dissatisfaction despite relatively good theoretical awareness of healthy eating. In our sample, 8.6% of students reported disordered eating or a history of eating disorders, which is lower than the 4–32% prevalence of high eating disorder risk and the 18% lifetime prevalence of eating disorders reported in previous studies of nutrition and dietetics students [31,32,33]. However, this difference should be interpreted with caution, as our study relied solely on self-reported history rather than validated screening tools assessing current risk or symptoms, and some students may therefore have chosen not to disclose these problems. At the same time, 25.3% of our students reported a goal of fat reduction, which may reflect some degree of body dissatisfaction, although this proportion is still lower than in earlier studies, where 37–86% of students reported body image or fat dissatisfaction, 66–68% wished to be thinner or weigh less, and one study found body-weight dissatisfaction in all participants [34,35,36,37]. University life is often associated with time pressure, irregular daily schedules, emotional stress, independent food choices, and greater reliance on convenience foods, all of which may contribute to a lower intake of fruits, vegetables, dairy, and fish, and therefore to lower intake of several nutrients [31,38]. In this context, our baseline results do not necessarily contradict the students’ field of study but rather highlight the gap between nutrition knowledge and actual dietary behaviour under real-life academic conditions.

The observed sex differences should also be interpreted carefully. Men consumed significantly higher amounts of several nutrients than women, especially energy, protein, fat, carbohydrates, sodium, potassium, magnesium, zinc, iodine, and vitamin C. However, this likely reflects a higher overall energy intake rather than a clearly healthier or more nutrient-dense diet. Similar sex-related differences in food intake among university students have been described elsewhere, with men often reporting a higher absolute intake but not necessarily better diet quality [32,39]. Therefore, absolute nutrient intake should be interpreted in the context of total energy intake and overall dietary pattern rather than in isolation.

The most important finding of the present study is the improvement in dietary intake observed during the semester. The largest changes were seen in carbohydrate and fibre intake, followed by marked increases in potassium, vitamin C, and magnesium, as well as smaller but still significant increases in iodine, zinc, vitamin K, vitamin E, iron, monounsaturated fatty acids, and total energy intake. At the same time, sodium and saturated fat intake decreased. Sodium intake should, however, be interpreted with caution, as the analysis may not have fully captured table salt added during cooking or at the table. The decrease in saturated fat may reflect improved understanding of fat quality and healthier food choices during the semester. Taken together, this pattern suggests not only a quantitative increase in food intake but also a qualitative shift towards a more favourable dietary profile. In practical terms, this may reflect higher consumption of fruit, vegetables, legumes, nuts, and whole grains, together with lower reliance on highly processed foods, which would be consistent with the observed increases in fibre and several micronutrients and the decreases in sodium and saturated fat.

This interpretation is supported by previous studies showing that nutrition education or curriculum-related exposure can improve selected dietary behaviours among university students, particularly food-choice quality rather than anthropometric outcomes. López-Moreno et al. (2023) found that a one-year nutrition education course for health science students was associated with improvements in dietary behaviour, including greater consumption of vegetables, legumes, nuts, and fish, as well as favourable changes in certain cardiometabolic markers [40]. Similarly, Helbach et al. (2023) reported improved adherence to dietary guidelines, particularly for fruit and vegetable intake, after a low-threshold nutrition lecture series among German medical students [41]. More broadly, a recent systematic review concluded that the higher-education nutrition interventions can improve dietary quality and food-related behaviours, although the strength of the evidence remains heterogeneous and strongly dependent on intervention design and implementation [42]. A systematic review by Deliens et al. (2016) [7] also evaluated different educational programmes aimed at improving dietary habits among university students and concluded that such interventions are generally effective. However, these programmes focused mainly on fruit and vegetable intake, while fewer studies examined effects on macronutrient, micronutrient, or total energy intake [7]. Among those that did assess macronutrients, some reported results opposite to ours, such as reductions in total energy and carbohydrate intake [43], whereas others found no significant differences [44,45]. Data specifically focused on dietary intake assessment in nutrition and dietetics students remains limited. Therefore, our findings support the idea that even routine academic exposure to nutrition content may positively influence food choices over a relatively short period.

At the same time, the increase in total energy intake in our study needs careful interpretation. It would be too simple to describe this change as either favourable or unfavourable without considering the full nutrient pattern. On the one hand, a higher total energy intake may be a concern if considered alone. On the other hand, the simultaneous increase in fibre, potassium, magnesium, vitamin C, and monounsaturated fatty acids, together with a lower sodium and saturated fat intake, suggests that at least part of this increase may have been accompanied by better nutrient density and better food quality. It is also possible that some of the apparent increase reflects more complete or more accurate dietary recording at follow-up, as students were trained throughout the semester to create food records and meal plans. Self-reported dietary tools are known to be vulnerable to misreporting, including underreporting at baseline and greater recording awareness over time, especially in individuals with increasing nutrition knowledge [12,46].

The marked increase in fibre intake is particularly noteworthy because low fibre intake was one of the clearest baseline inadequacies. From a nutritional point of view, this is one of the most meaningful findings, since higher fibre intake is generally associated with better overall diet quality and may also be linked to better digestion, greater satiety, prevention of cardiovascular disease, and improved mental well-being, although causal mechanisms are still being studied [47].

Nevertheless, the persistence of a low intake of some nutrients at baseline, and possibly still insufficient intake in some students at follow-up, remains important. Vitamin D, iodine, and calcium are nutrients for which adequacy may be difficult to achieve through spontaneous dietary improvement alone, especially in populations with low intake of fortified foods, fish, dairy products, or supplements. Similar results were reported by Farhat et al. (2019), who found low intake of calcium, vitamins A and D, magnesium, potassium, iodine, and selenium in young adults aged 18–25 years [48]. This is particularly relevant in Central European settings, where vitamin D intake from food is commonly low and sunlight-dependent synthesis varies across seasons [49,50]. Our data, therefore, suggest that general nutrition education may improve overall diet quality but may be less effective at correcting nutrient deficiencies that require more specific food choices, food fortification, or supplementation.

The absence of significant changes in body composition despite improved dietary intake is not unexpected and should not be seen as contradictory. First, the study period was relatively short, and participants were young adults without a targeted weight-loss, muscle-gain, or exercise intervention. In this context, moderate dietary changes over one semester may not be enough to produce measurable changes in fat mass or skeletal muscle mass. Second, body composition is influenced not only by diet, but also by total energy balance, physical activity, sedentary behaviour, sleep, stress, hormonal status, and baseline adiposity. This interpretation is supported by López-Moreno et al. (2023) [40], who observed improvements in dietary behaviour after nutrition education without significant changes in BMI, fat mass, or waist-to-hip ratio. Our results, therefore, agree with previous evidence suggesting that nutrition education may affect food choices sooner than anthropometric or body-composition outcomes [40].

The ANCOVA findings are especially relevant in this respect as they suggest that follow-up body fat percentage was determined mainly by baseline body fat percentage, while changes in reported energy and protein intake were not significant predictors. This is physiologically plausible. In relatively healthy young adults over a short observation period, baseline adiposity often remains the strongest predictor of later adiposity, whereas moderate fluctuations in self-reported diet may not explain enough variation to produce statistically detectable effects. The absence of an independent effect of increased protein intake is also understandable, since protein intake was already relatively high at baseline. Once protein intake is adequate or above recommended levels, further short-term variation may have limited importance for adiposity, especially in the absence of structured resistance training.

Another important factor is the academic context in which the follow-up assessment took place. A substantial part of the semester likely coincided with increasing academic pressure, including examination periods and, for some students, preparation for state examinations. This may have affected both dietary intake and energy expenditure in ways that are difficult to separate. Recent evidence shows that exam periods in university students may be associated with a poorer diet quality, lower physical activity, greater sedentary behaviour, increased fat mass, and reduced muscle mass [51]. Other studies and reviews have similarly shown that stress and anxiety are associated with a poorer diet quality, more emotional eating, and less healthy eating patterns in student populations [52,53]. At the same time, academic stress does not affect all students in the same way: in some students, it may reduce appetite, while in others, it may increase snacking, emotional eating, total energy intake, and preference for easily available foods. Therefore, the dietary changes observed in our study may reflect not only the effect of education but also a combination of academic exposure, self-monitoring, stress-related coping, and changes in daily routine.

This may also help explain why dietary quality improved while body composition remained unchanged. During academically demanding periods, students may make somewhat better food choices due to their nutrition education, but at the same time, consume more total energy and move less. This mixed effect could partly explain the pattern observed in our study.

Another factor that deserves attention is the possible influence of the psychological burden, which was not directly measured in the present study. This is an important limitation because psychological stress may strongly affect appetite regulation, food preferences, meal timing, sleep, motivation to move, and adherence to dietary recommendations. Emotional eating has been shown to correlate with perceived stress and barriers to healthy eating among college students, particularly in individuals using more avoidant coping strategies [52]. Thus, psychobehavioural influences may have affected both dietary and body-composition trajectories in our sample and may partly explain some of the unexplained variability in the results. In addition, lifestyle-related factors such as physical activity, smoking, and alcohol consumption—although collected—were not analysed in relation to dietary changes in the present study but may have contributed to the observed outcomes. These factors will be addressed in more detail in future research using validated assessment tools.

### 4.1. Strengths and Limitations

The present study has several strengths. It used a prospective within-subject design, assessed both dietary intake and body composition at two time points, and captured a broad range of macro- and micronutrients rather than only selected variables. Another strength is that the direction of change across nutrients was internally consistent, which supports the plausibility of a real behavioural shift rather than isolated statistical findings. The study also focused on a specific and relevant population—Czech nutrition students—among whom the relationship between theoretical knowledge and actual behaviour is of particular interest. In addition, the repeated assessment across the academic semester allowed observation of changes under real-life university conditions rather than under tightly controlled laboratory conditions.

However, several limitations of this study should be acknowledged. First, dietary intake was assessed using self-reported 3-day food records. Although this method is widely used in nutrition research, it remains prone to underreporting, selective omission, reactivity, and a limited ability to reflect habitual intake, particularly for episodically consumed foods and micronutrients [12,46]. In addition, the relatively short recording period may not have fully captured usual dietary intake across the semester. Dietary intake and nutrient consumption may also have been influenced by seasonality, as the period during which dietary records were collected could have affected food availability, food choice, and the consumption of seasonal products [54,55]. Nutritional analysis also depended on the software used, including the quality, completeness, up-to-date status, and food-matching procedures of the nutrient database. As a result, software-based nutrient calculations may introduce additional uncertainty, particularly for micronutrients, mixed dishes, recipe decomposition, and supplement use [16,56].

Second, body composition was measured using multi-frequency bioelectrical impedance analysis rather than a criterion method such as dual-energy X-ray absorptiometry. Although BIA is practical and suitable for field-based research, its estimates can be affected by hydration status, recent food and fluid intake, recent physical activity, and other acute physiological fluctuations [57]. Even with standardized measurement procedures, such sources of variability cannot be fully eliminated under free-living conditions. Body composition estimates may also have been influenced by behaviour on the preceding day, including unusually high physical activity, atypical food intake, late eating, or fluid shifts. As the sample consisted predominantly of women, another relevant consideration is menstrual-cycle-related variability. Although recent evidence suggests that the menstrual cycle phase may have only a limited effect on BIA-derived body composition estimates under controlled conditions, cycle-related changes in appetite, water retention, mood, fatigue, and physical activity may still have contributed to variability in repeated field measurements and self-reported dietary intake [58].

Third, several potentially important contextual factors were not assessed. Psychological state, stress load, and coping style were not measured, despite their likely relevance for both dietary behaviour and energy expenditure. In addition, physical activity was assessed using a non-validated self-reported questionnaire based on reported type, weekly frequency and duration.

Further limitations relate to the study sample. The male subgroup was relatively small, which reduced statistical power for sex-stratified analyses and may have increased the instability of some sex-specific findings. In addition, the findings have limited generalisability because the sample comprised Czech university students enrolled in a nutrition-related programme. Dietary habits, food environments, and educational contexts may differ across countries; therefore, the results may not be directly transferable to other student populations. Furthermore, as the study did not include a non-nutrition control group, causal inferences should be made with caution, as the observed changes cannot be attributed solely to the educational exposure.

A further limitation concerns participant attrition. A formal comparison between included and excluded participants was limited by the availability of baseline data for excluded individuals. Incomplete dietary records and missing body composition measurements were among the primary reasons for exclusion, which restricted the possibility of assessing differences in key variables such as dietary intake and body composition. Participants were not excluded based on their baseline values or study outcomes; rather, exclusions were primarily due to logistical and compliance-related factors, including inability to attend the second measurement, incomplete records, or personal circumstances (e.g., study discontinuation or study abroad). Nevertheless, some degree of selection bias cannot be ruled out.

### 4.2. Implications and Future Directions

Taken together, these findings suggest that nutrition-related education may improve dietary quality in the short term, but such changes may not be sufficient to produce measurable changes in body composition within one semester. A practical implication is that university nutrition education may serve as an important early preventive tool by improving food choice and health awareness during a period when lifelong habits are being formed.

However, longer longitudinal designs, ideally spanning one or more academic years, are needed to determine whether these dietary improvements are maintained and whether delayed effects on body composition emerge over time. In addition, consideration of individual variability in response to dietary changes, including the analysis of individual trajectories and potential determinants of response, may provide further insight into the effectiveness of such educational exposure.

Future studies should also include repeated assessment of key behavioural factors using standardized tools, such as the International Physical Activity Questionnaire (IPAQ) for physical activity, the Perceived Stress Scale-10 (PSS-10) for perceived stress, and the Pittsburgh Sleep Quality Index (PSQI) for sleep quality, as these factors are highly relevant in student populations and may influence diet-related outcomes. In addition, dietary intake could be assessed more comprehensively using a Food Frequency Questionnaire (FFQ), which may provide a more robust estimate of habitual dietary intake, food preferences, and longer-term eating patterns. Furthermore, future research should incorporate direct assessment of nutritional knowledge (e.g., knowledge of dietary recommendations and healthy eating principles), as this would allow for a better understanding of the relationship between knowledge and actual dietary behaviour.

## 5. Conclusions

In conclusion, one semester of nutrition-related education was associated with an improved dietary intake among nutrition students, particularly in fibre, selected micronutrients, sodium, and saturated fat. However, no significant short-term changes in body composition were observed. The findings suggest that nutrition education may contribute to healthier dietary behaviour and could play a role in preventive healthcare among young adults, although body composition is likely shaped by a broader range of lifestyle and contextual factors.

## Figures and Tables

**Table 1:** Baseline nutrient intake of the study population, comparison between women and men, and mean intake expressed as a percentage of D-A-CH dietary reference values (*n* = 102; women = 80, men = 22).

Nutrient	Whole Sample (Mean ± SD)	Women (Mean ± SD)	Men (Mean ± SD)	*p* Value	D-A-CH Reference (w/m)	Women (% of D-A-CH Reference)	Men (% of D-A-CH Reference)
Energy (kcal/day)	2114 ± 632	1911 ± 370	2853 ± 811	<0.001	2200/2800	86.8	101.9
Protein (g/day)	107.2 ± 45.1	91.4 ± 23.4	164.6 ± 57.1	<0.001	48/57	190.5	288.7
Fat (g/day)	80.7 ± 26.6	74.1 ± 18.6	104.7 ± 35.8	<0.001	73.3/93.3	101.0	112.2
Carbohydrates (g/day)	241.4 ± 69.6	222.2 ± 46.8	311.3 ± 90.9	<0.001	337/433	65.9	71.9
Fibre (g/day)	15.5 ± 8.5	15.1 ± 8.2	16.6 ± 9.2	0.39	30	50.3	55.8
Cholesterol (mg/day)	348 ± 276	260 ± 169	667 ± 345	<0.001	<300	86.7	222.5
Sodium (mg/day)	1346 ± 907	1186 ± 712	1929 ± 1242	<0.001	1500	79.1	128.6
Calcium (mg/day)	630 ± 372	571 ± 302	847 ± 500	0.003	1000	57.1	84.7
Iron (mg/day)	10.14 ± 4.82	9.28 ± 4.17	13.25 ± 5.66	0.002	16/11	58.0	120.5
Vitamin C (mg/day)	90.9 ± 49.8	83.1 ± 47.5	118.9 ± 47.9	0.004	95/110	87.5	108.1
Vitamin D (µg/day)	2.31 ± 3.01	2.14 ± 2.82	2.92 ± 3.56	0.28	20	10.7	14.6
Vitamin K (µg/day)	208 ± 285	219 ± 294	165 ± 242	0.37	60/70	365.7	235.2
Potassium (mg/day)	2013 ± 954	1795 ± 901	2806 ± 684	<0.001	4000	44.9	70.1
Vitamin B_12_ (µg/day)	6.27 ± 42.30	1.91 ± 3.54	22.09 ± 89.06	0.08	4	47.8	552.3
Folate (µg/day)	328 ± 930	219 ± 270	725 ± 1884	0.09	300	73.3	240
Magnesium (mg/day)	234 ± 124	208 ± 106	331 ± 138	<0.001	300/350	69.2	94.5
Zinc (mg/day)	5.24 ± 3.25	4.85 ± 3.12	6.69 ± 3.28	0.01	7/11	69.3	60.8
Iodine (µg/day)	63.5 ± 69.8	50.2 ± 46.9	111.9 ± 107.5	<0.001	150	33.5	74.6
Vitamin E (mg/day)	6.41 ± 3.98	5.67 ± 3.27	9.11 ± 5.01	0.002	8	70.9	113.9

Abbreviations: SD—standard deviation; w—women; m—men.

**Table 2:** Changes in dietary intake and micronutrients (*n* ≈ 101–102).

Nutrient	*n*	Mean Change	Cohen’s d	*p* (FDR)	% Improved
Carbohydrates (g/day)	102	54.2	1.07	<0.001	86.3
Fibre (g/day)	102	9.33	1.04	<0.001	83.3
Potassium (mg/day)	102	766	0.95	<0.001	80.4
Vitamin C (mg/day)	101	69.2	0.86	<0.001	83.2
Magnesium (mg/day)	102	86.2	0.73	<0.001	76.5
MUFA (g/day)	102	4.17	0.59	<0.001	72.5
Energy (kcal/day)	102	219	0.58	<0.001	72.5
Iodine (µg/day)	102	24.0	0.4	<0.001	70.6
Zinc (mg/day)	102	2.10	0.34	0.001	66.7
Vitamin K (µg/day)	102	116	0.29	0.005	66.7
Vitamin E (mg/day)	102	5.14	0.27	0.01	75.5
Iron (mg/day)	102	8.43	0.24	0.019	83.3
Sodium (mg/day)	102	−221	−0.26	0.011	53.9
Saturated fat (g/day)	102	−2.04	−0.24	0.019	52.0

Abbreviations: MUFA—monounsaturated fatty acids; FDR—false discovery rate. Note: Positive values indicate increases from baseline to follow-up. Vitamin C was not statistically evaluated in one subject due to abnormal values.

**Table 3:** Baseline body composition characteristics of the study participants (*n* = 102; women *n* = 80; men *n* = 22). Values are presented as mean ± SD.

Variable	Total Sample	Women	Men	*p*-Value
Age (years)	22.7 ± 1.5	22.7 ± 1.4	23.0 ± 1.6	0.366
Height (cm)	170.9 ± 10.2	167.1 ± 6.9	184.9 ± 7.7	<0.001
Weight (kg)	69.2 ± 14.6	64.1 ± 9.9	87.6 ± 14.0	<0.001
BMI (kg/m^2^)	23.5 ± 3.1	22.9 ± 3.0	25.5 ± 2.7	<0.001
Body Fat Mass (kg)	15.9 ± 6.7	17.1 ± 6.6	11.7 ± 5.3	<0.001
Percent Body Fat (%)	23.3 ± 8.7	26.1 ± 7.3	13.0 ± 4.9	<0.001
Skeletal Muscle Mass (kg)	29.7 ± 8.6	25.9 ± 3.8	43.6 ± 6.8	<0.001
Basal Metabolic Rate (kcal/day)	1521 ± 306	1386 ± 137	2011 ± 247	<0.001
Waist–Hip Ratio	0.85 ± 0.05	0.85 ± 0.05	0.85 ± 0.06	1.000
Visceral Fat Area	68.3 ± 33.7	73.5 ± 33.7	49.7 ± 27.0	0.001
InBody Score	80.3 ± 9.0	77.4 ± 6.7	90.7 ± 8.8	<0.001
Total Body Water (L)	38.9 ± 10.3	34.4 ± 4.6	55. 6 ± 8.3	<0.001
Bone Mineral Content (kg)	3.15 ± 0.82	2.81 ± 0.41	4.39 ± 0.76	<0.001

**Table 4:** ANCOVA predicting follow-up body fat percentage (*n* = 102).

Predictor	β (Unstandardized)	SE	t	*p*-Value	95% CI
Intercept	−3.09	3.230	−0.96	0.340	−9.50, 3.31
Baseline PBF (%)	0.927	0.031	30.11	<0.001	0.866, 0.988
Δ Protein (g/day)	−0.005	0.010	−0.53	0.599	−0.025, 0.014
Δ Energy (kcal/day)	0.0006	0.001	0.81	0.420	−0.001, 0.002
Age (years)	0.221	0.140	1.57	0.119	−0.058, 0.499
Sex	−0.885	0.639	−1.38	0.170	−2.153, 0.384

Model statistics: R^2^ = 0.943; adjusted R^2^ = 0.940; F (5.99) = 325.1; *p* < 0.001. Abbreviation: PBF—percent body fat.

## Data Availability

The raw data supporting the conclusions of this article will be made available by the authors on request. Due to the nature of the data and to ensure responsible use and proper interpretation, the datasets are not publicly available. The main raw data was provided during the submission.

## Supplementary Materials

The following supporting information can be downloaded at: https://www.mdpi.com/article/10.3390/healthcare14101258/s1.

## Abbreviations

The following abbreviations are used in this manuscript:

BIA	Bioelectrical impedance analysis
MF-BIA	Multi-frequency bioelectrical impedance analysis
DXA	Dual-energy X-ray absorptiometry
SD	Standard deviation
IQR	Interquartile range
FDR	False discovery rate
ANCOVA	Analysis of covariance
BMI	Body mass index
PBF	Percent body fat
MUFA	Monounsaturated fatty acids
D-A-CH	German-Austrian-Swiss dietary reference values
IPAQ	International Physical Activity Questionnaire
PSS-10	Perceived Stress Scale-10
PSQI	Pittsburgh Sleep Quality Index
